# The optimal analgesic method in saline infusion sonogram: A comparison of two effective techniques with placebo

**DOI:** 10.4274/tjod.46667

**Published:** 2016-09-15

**Authors:** Sadullah Özkan, Bülent Kars, Önder Sakin, Aylin Onan Yılmaz, Yaren Tuba Bektaş, Halim Ömer Kaşıkçı

**Affiliations:** 1 Dr. Lütfi Kırdar Training and Research Hospital, Clinic of Obstetrics and Gynecology, İstanbul, Turkey; 2 Dr. Lütfi Kırdar Training and Research Hospital, Clinic of Family Medicine, İstanbul, Turkey

**Keywords:** Saline infusion sonography, pain, topical anesthesia, lidocaine

## Abstract

**Objective:**

Operations performed with local anesthesia can sometimes be extremely painful and uncomfortable for patients. Our aim was to investigate the optimal analgesic method in saline infusion sonograms.

**Materials and Methods:**

This study was performed in our Clinic of Obstetrics and Gynecology between March and August 2011. Ninety-six patients were included. Patients were randomly divided into groups that received saline (controls, group 1), paracervical block (group 2), or paracervical block + intrauterine lidocaine (group 3). In all groups, a visual analogue scale score was performed during the tenaculum placement, while saline was administered, and 30 minutes after the procedure.

**Results:**

When all the patients were evaluated, the difference in the visual analogue scale scores in premenopausal patients during tenaculum placement, during the saline infusion into the cavity, and 30 minutes following the saline infusion sonography were statistically different between the saline and paracervical block groups, and between the saline and paracervical block + intrauterine lidocaine group. However, there was no statistically significant difference between paracervical block and paracervical block + intrauterine lidocaine groups.

**Conclusion:**

As a result of our study, paracervical block is a safe method to use in premenopausal patients to prevent pain during saline infusion sonography. The addition of intrauterine lidocaine to the paracervical block does not increase the analgesic effect; moreover, it increases the cost and time that the patient stays in the dorsolithotomy position by 3 minutes.

## INTRODUCTION

There is a necessity to evaluate the endometrial cavity in many different gynecologic conditions. Pre and post-menopausal bleeding, endometrial lesions found with ultrasound, and evaluation of endometrial cavity before performing hysterectomy are some indications that may require saline infusion sonography (SIS)^(1,2)^.

The specificity and sensitivity of SIS for detecting endometrial pathology were 81-100% and 85-100%, respectively. For detecting submucosal myoma, it has sensitivity of 57-100% and specificity of 96-100%. For detecting endometrial hyperplasia or cancer it has sensitivity of 29-80% and specificity of 82-100%^(3)^. In sum, SIS is a valuable and indispensable method in gynecology practice.

Unfortunately, SIS may cause pain and discomfort depending on the technique and methods of anesthesia. Grasping the cervix with a tenaculum, movement of cannulas in the uterus, and distention of the uterine cavity with saline may cause pain in the procedure. Hence, active cooperation of patients is highly desirable to obtain maximum efficacy, and effective anesthesia becomes very important. Paracervical block (PCB) is the most frequently used method to prevent pain in the procedure. Previous reports showed that intrauterine lidocaine (IUL) is also a safe and effective method for preventing pain in outpatient gynecologic procedures^(4,5,6,7,8,9)^.

We designed a randomized controlled trial to compare the efficacy of PCB vs. IUL, and also with placebo.

## MATERIALS AND METHODS

We conducted this study between March 2011 and August 2011 in a tertiary reference center. The study was approved by our ethical committee. All participants gave their written informed consent for the study. We included 120 women who underwent SIS for various reasons. We excluded women with severe systemic medical conditions such as heart failure and uncontrolled severe hypertension, and cervical stenosis, acute cervicitis and/or vaginitis, and lidocaine allergy. The remaining 96 women were randomized into three groups: saline controls (group 1), PCB (group 2), and PCB + IUL (group 3); randomization was performed using computer-generated random number tables.

We collected data about patient characteristics including age, gravidity, parity, history of abortion, any known allergy, current drug use, and medical and gynecologic history from patient records.

All women underwent a bimanual pelvic examination to determine the size and position of the uterus. The cervix was exposed using a bivalve speculum and washed with povidone-iodine solution. In the PCB group, 2 mL 2% lidocaine (Iekaine Ampoule, IE Ulagay, İstanbul, Turkey) was injected into the cervix at 4- and 8 o’clock positions at a depth of 2-3 cm after confirming the tip of needle was not inside a vessel lumen. Five minutes were allowed to pass to ensure the anesthetic effect of lidocaine had started. In the PCB + IUL group, an 18-gauge intravenous catheter was gently inserted into the cervical canal up to the internal os. Two milliliters of 2% lidocaine was injected into the uterine cavity. Again, 5 minutes were allowed to pass to ensure that the anesthetic effect of the lidocaine had begun. All forms of anesthetic methods were applied before grasping the cervix with a tenaculum. We used no anesthetic in the control group. Two milliliters of 0.9% saline solution was injected into the cervix at 4- and 8 o’clock positions instead of lidocaine. Five minutes were allowed to pass to create similar circumstances with the other groups. The cervix was grasped with a tenaculum at 11- and 1 o’clock positions. A number 4 carmen cannula was inserted in the uterine cavity. The uterine cavity was filled with 50 mL of normal saline solution. The same operator performed all SIS procedures in the same way with help of the same nurse. Therefore, other variables that may affect pain score were controlled. A tenaculum was used in all patients in the standard procedure technique. Difficulty during passing the cervix and SIS findings were not recorded in our study.

We evaluated pain scores using a 10-cm visual analogue scale (VAS), where 0 cm represented no pain and 10 cm represented worst pain imaginable. We evaluated pain scores at three different points: Immediately after installation of normal saline, at the end of the procedure, and 30 minutes after the procedure. All patients were prescribed 500 mg azithromycin as prophylaxis.

Statistical calculations were performed using the Statistics Package for the Social Sciences (SPSS) for Windows version 13.0. Descriptive data are presented as mean ± standard deviation or standard error. One-way ANOVA and Post-hoc Tukey tests were used to compare parametric variables and to compare differences between groups, respectively. A value of p<0.05 was accepted as statistically significant.

## RESULTS

The ages of the 96 patients who participated in the study ranged from 23 to 62 years. The mean age of group 1 was 38.38±7.48 years, group 2 was 35.25±8.08 years, group 3 was 37.03±7.27 years. There was no statistically significant difference between the mean ages of the groups (p>0.05).

Of the patients included in the study, 16 were postmenopausal and 80 were premenopausal; group 1 (n=32) 26 premenopausal, 6 postmenopausal patients; group 2 (n=32) 27 premenopausal, 5 postmenopausal; and group 3 (n=32) 27 premenopausal, 5 postmenopausal patients.

The median scores of the groups were gravida (2, 3, 3), living child (2, 2, 2), abortion (0, 0, 0), and curettage (0, 0, 0) respectively. It has been found to disperse in accordance with the average of all these groups.

We found significant differences between groups at tenaculum placement. We used the Post-hoc Tukey test to determine which group had the statistically significant score. We found that pain scores were significantly higher in the control group (p=0.002), but there was no significant difference between either study group (p=0.596).

After the injection of sterile saline solution, the control group had significantly increased pain scores, different from both study groups (p=0.045). We found no significant difference between either study group at this point (p=0.835). During tenaculum use, the mean pain in the group 1 was 27.40±25.58, group 2 was 21.74±23.25, and group 3 was 11.74±11.54 (Graphic 1). During saline infusion, the mean pain in the group 1 was 25.20±27.66, group 2 was 29.12±14.56, and group 3 was 20.63±19.50 (Graphic 2) (Table 1, 2, 3).

## DISCUSSION

Patients experience pain in gynecologic outpatient diagnostic interventions. We aimed to determine whether it was correct to use different anesthetic methods in daily clinical practice, and thus we compared PCB and PCB + IUL with placebo.

In the study of Guney et al.^(5)^, IUL that was applied just after buccal misoprostol was found effective preventing pain. IUL failed to prevent pain in procedures such as endometrial biopsy or hysterosalpingograhy in other studies^(6,7)^. Guney et al.^(5)^ attributed this difference to the combined use of lidocaine with other drugs. Though there was no significant difference between study groups, we also find that lidocaine decreased pain with statistical significance. The reason of this result may be the limited local effect of lidocaine. Patients feel pain the most at the time of grasping the cervix with a tenaculum and the insertion of a carmen cannula. Lidocaine shows its anesthetic effect through free nerve endings as described in previous studies. Guney et al.^(5)^ found that pain was decreased in their IUL group during endometrial curettage. We did not perform endometrial biopsy, instead only the uterine cavity was distended in our study. We found no significant differences, probably because we performed a less painful procedure than that of Guney et al.^(5)^.

PCB and PCB + IUL were effective at preventing pain in all premenopausal women in our study. The same effect could not be shown in postmenopausal women. To our knowledge, no studies have compared pain scores of women according to their menopausal status. Only Guney et al.^(5)^ noted that combined use of IUL and misoprostol was effective at preventing pain in premenopausal women, whereas it was not effective in postmenopausal women. More randomized studies are warranted to determine which anesthetic method would be appropriate for postmenopausal women in outpatient gynecologic procedures.

Van den Bosch et al.^(8)^ compared gel infusion sonography with SIS in their study of 2009. They found both methods were similar in use but pain caused by the procedure was heightened in SIS. They attributed this difference to the lubricant effect of gel, which made it easier to pass the instrument through the cervix. In their second study, they compared gel infusion sonography alone with gel infusion sonography plus IUL. The authors could not show significant differences in the mean VAS scores. Performing SIS is much easier than gel infusion sonography in outpatient settings. Moreover, the long-term effect of gel use remains unknown^(9)^.

The effect of different anesthetic methods on endometrial curettage, hysterosalpingograhy, and hysteroscopy has been extensively studied. The results are conflicting because of the different natures of the procedures. We think our study will help those who need an effective method to prevent pain in SIS.

## CONCLUSION

In conclusion, paracervical block is effective at preventing pain in premenopausal women undergoing SIS. The addition of IUL to PCB does not decrease pain but increases both the time and cost of the procedure.

## Figures and Tables

**Table 1:**
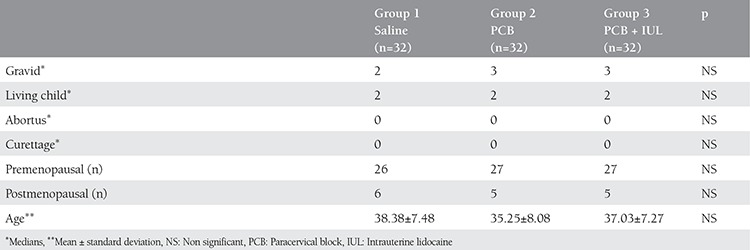
Demographic characteristics of the groups

**Table 2:**
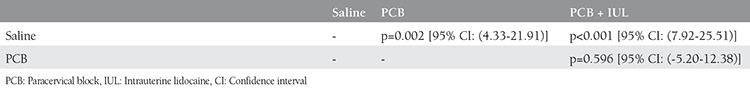
The visual analogue scale scores of all patients during tenaculum use during saline infusion sonogram

**Table 3:**
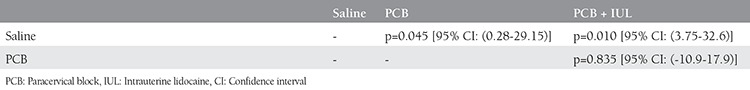
Visual analogue scale scores of all patients during saline infusion into the uterine cavity

**Graphic 1:**
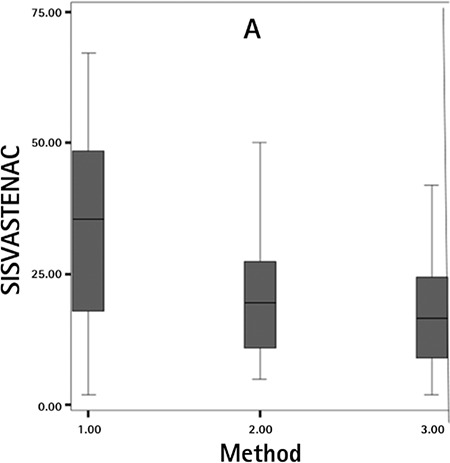
Graph showing the mean ± standard deviation D values of pain visual analogue scale scores during tenaculum application *SIS: Saline infusion sonography, VAS: Visual analogue scale,* *TENAC: Tenaculum*

**Graphic 2:**
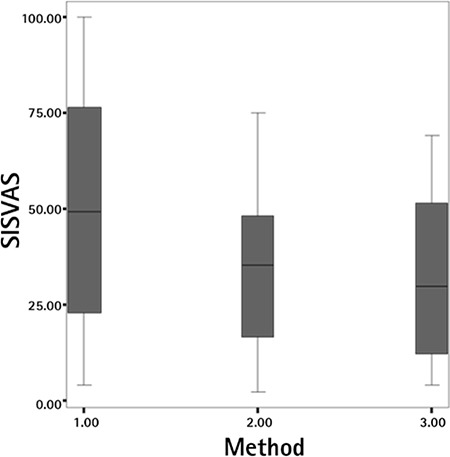
The graph showing the mean ± standard deviation values of pain visual analogue scale scores during saline infusion into the uterine cavity *SIS: Saline infusion sonography, VAS: Visual analogue scale*
